# Antidote for Strychnine

**Published:** 1855-12

**Authors:** 


*Antidote for Strychnine.*—A writer in the American Journal of Medical Sciences, speaks confidently of the efficacy of simple cerate or lard, as an antidote for strychnine.

